# Free-Breathing Phase-Resolved Oxygen-Enhanced Pulmonary MRI Based on 3D Stack-of-Stars UTE Sequence

**DOI:** 10.3390/s22093270

**Published:** 2022-04-24

**Authors:** Pengfei Xu, Jichang Zhang, Zhen Nan, Thomas Meersmann, Chengbo Wang

**Affiliations:** 1 Electrical and Electronic Engineering, Faculty of Science and Engineering, University of Nottingham Ningbo China, Ningbo 315100, China; pengfei.xu2@nottingham.edu.cn (P.X.); jichang.zhang@nottingham.edu.cn (J.Z.); zhen.nan2@nottingham.edu.cn (Z.N.); 2 Sir Peter Mansfield Magnetic Imaging Center, University of Nottingham, Nottingham NG7 2RD, UK; thomas.meersmann@nottingham.ac.uk; 3 Nottingham Ningbo China Beacons of Excellence Research and Innovation Institute, Ningbo 315040, China

**Keywords:** oxygen-enhanced lung MRI, phase resolve, free-breathing, UTE sequence, stack-of-stars

## Abstract

Compared with hyperpolarized noble gas MRI, oxygen-enhanced lung imaging is a cost-effective approach to investigate lung function. In this study, we investigated the feasibility of free-breathing phase-resolved oxygen-enhanced pulmonary MRI based on a 3D stack-of-stars ultra-short echo time (UTE) sequence. We conducted both computer simulation and in vivo experiments and calculated percent signal enhancement maps of four different respiratory phases on four healthy volunteers from the end of expiration to the end of inspiration. The phantom experiment was implemented to verify simulation results. The respiratory phase was segmented based on the extracted respiratory signal and sliding window reconstruction, providing phase-resolved pulmonary MRI. Demons registration algorithm was applied to compensate for respiratory motion. The mean percent signal enhancement of the average phase increases from anterior to posterior region, matching previous literature. More details of pulmonary tissues were observed on post-oxygen inhalation images through the phase-resolved technique. Phase-resolved UTE pulmonary MRI shows the potential as a valuable method for oxygen-enhanced MRI that enables the investigation of lung ventilation on middle states of the respiratory cycle.

## 1. Introduction

MRI is a non-invasive imaging modality to produce 2D or 3D anatomical images without radiation damage. It has been widely used to image many organs, such as brain, kidney and liver at variable magnetic fields [1,2]. However, the traditional MRI does not perform well to image the lung partially due to the special pulmonary structure. Numerous air–tissue interfaces give rise to inhomogeneities in the local magnetic field which further lead to reduced transverse relaxation time, T2* (1–2 ms), resulting in rare signal collected before it is decayed [1,3,4]. Furthermore, inherently low proton density brings large challenges to obtain enough MRI signals for anatomic imaging.

In addition to conventional proton MRI, multinuclear techniques that use Fluoride-containing gas, hyperpolarized ^131/129^Xe or ^83^Kr as gaseous imaging contrast agents are being developed to investigate lung functions in healthy subjects and patients with various lung diseases [5,6,7]. Hyperpolarized noble gas (^129^Xe and ^3^He) is able to generate strong signals because of its extremely high polarization [7,8], which is as much as 10^5^ times greater than its thermal equilibrium polarization [9]. The pulmonary ventilation and diffusion can be easily investigated, since the gas diffuses much faster than protons in water. Nevertheless, the multinuclear technique needs a polarizer to generate hyperpolarized gas and extra accessories of current scanners, such as specific coils and broadband transceiver hardware, which increases difficulties to apply this technique in clinic. 

The oxygen molecules are not only involved in gas exchange in the alveoli but also dissolve into the blood as well, accelerating the longitudinal relaxation of nearby tissues due to the paramagnetism property of oxygen. The oxygen molecules can reduce the T1 value reported firstly by Young et al. [10] and visually illustrated on excised rat lungs by Goodrich et al. in 1991 [11]. Later, Edelman et al. investigated the possibility of employing pure oxygen to human lungs as contrast agents and observed a significant signal increase, approximately 50% [12]. Therefore, oxygen-enhanced lung MRI is expected to provide both ventilation and perfusion information of the lungs. Moreover, oxygen is readily available in clinical settings, which enables this technique to be more accessible for clinical use. Current primary imaging sequences for OE MRI include inversion recovery half Fourier-acquired single-shot turbo spin-echo (IR-HASTE) and ultra-short TE (UTE) MRI. For IR-HASTE sequence, the long echo train leads to the highly decayed signal of last several echoes [13]. On the other hand, the maximum percent signal enhancement (PSE) occurs near the zero-crossing point of the longitudinal relaxation recovery curve, resulting in low signal intensity [14]. UTE MRI uses the ultra-short echo time of about 8~200 us, much shorter than the reported T2* (1~2 ms) of lung tissues, which can provide more details of lung tissues and vessels [15,16]. In OE-UTE-MRI, the signal enhancement depends on both TE and flip angle, where the PSE increases with shorter TE and optimized flip angle. However, a larger flip angle may not provide greater signal enhancement because the signal intensity decreases after the Ernst angle [17], reducing the signal-to-noise ratio (SNR). In this paper, we optimized the flip angle from simulation to phantom and healthy human.

Another challenge of pulmonary imaging is the motion effects generated from respiration and cardiac motion, especially in Cartesian k-space filling scheme which suffers serious motion artifacts [18,19]. However, the radial sampling scheme oversamples the central part of k-space where the most important signal components are located, so that it is relatively insensitive to motion artifacts, allowing free breathing during MRI scan [20,21]. With the golden angle radial stack-of-stars acquisition technique, fewer spokes are needed to further accelerate the image acquisition [22]. Moreover, the radial acquisition allows reconstructing images with arbitrary view groups as each view goes through the center of k-space, which enables us to separate the data along time and space dimensions. Symmetrical distribution of spokes occurs when the number of spokes matches one of the Fibonacci numbers [23].

Although the stack-of-stars technique improved image quality, soft blurring artifacts still appear on pulmonary images resulting from respiratory and cardiac motion. To eliminate soft blurring, respiratory gating is often applied during MRI scans [24,25,26]. Alternatively, the self-gated technique based on the radial acquisition scheme has been proposed to lock the respiratory phase at the end of expiration or inspiration by several groups [27]. With the self-gated technique, respiratory signal could be extracted from raw k-space data by principal components analysis (PCA) algorithm [28] without the help of external respiratory sensors, such as pneumatic bellows and optical sensors. In lung imaging, the lung volume changes at different respiratory phases, resulting in variable oxygen concentration in alveoli. The PSE and T1 measurement at the end of expiration and inspiration has been reported in previous literatures. However, there are rare research data about middle states of a respiratory cycle. Phase-resolved oxygen-enhanced MRI could segment respiratory cycle into several phases based on the extracted respiratory signal. Using sliding window technique, the minimum motion resolution along respiratory cycle can be reduced to the time to collect one group spokes ($N_{z}\times TR$, where $N_{z}$ is the number of steps in z-direction), providing highly accurate of phase segmentation [29]. 

In this study, we simulated mean PSE changes with flip angle using Bloch equation and further verified the simulation on both homemade T1 phantoms and healthy human. Finally, we investigated signal enhancement at both ends and middle phases of a respiratory cycle at optimized flip angle. Hence, we confirmed the trend of mean PSE change with flip angle from simulation by using in vivo experiment, and then illustrated the feasibility of the proposed self-gated free-breathing phase-resolved oxygen-enhanced pulmonary imaging method. The phase-resolved oxygen-enhanced MRI provided sharp details on both normoxia and hyperoxic images in lung vessels and diaphragm.

## 2. Materials and Methods

### 2.1. Study Population

Four healthy subjects (3 M, 1 F) were enrolled following a protocol approved by the Institutional Review Board and informed written consent was obtained from each subject. Prior to each MRI scan, all subjects underwent pulmonary function test (PFT) to establish baseline global lung function. The spirometry measurements include forced expiratory volume in 1 s (FEV1), forced vital capacity (FVC), forced expiratory flow at 75% of FVC (FEF75), forced expiratory flow at 25% and 75% of the pulmonary volume and Peak. Percentage predicted values of these measurements were calculated based on Global Lung Function Initiative (GLI) Network [30].

### 2.2. MR Pulse Sequence and Imaging

Stack-of-stars center-out golden-angle UTE pulse sequence was implemented in this study. Before the human experiment, the Bloch simulation with variable flip angle (1°~90°) was conducted in MATLAB2020a (Mathworks, Natick, MA, USA), assuming T1/T2* values reported in previous literatures [15,20,31]. Other parameters were assumed to match with that used in human scans. Two homemade phantoms representing lung T1 before and post oxygen breath were used to verify simulation and optimize flip angle. T1 phantoms were made by mixing GdCl_3_ and agarose with varying concentrations [32]. Before experiment, the inversion recovery spin echo (IRSE) sequence was used to measure T1 and T2 as the ground truth values, which are 1391.9 ± 4.4 ms and 1292.3 ± 7.4 ms for T1 and 83.68 ± 0.82 ms and 74.84 ± 1.89 ms for T2, respectively. The 7.2% and 10.6% reduction in T1 and T2 values were similar to the T1 and T2* reduction in lung tissues with air and alternative oxygen breathe [33]. 

For in vivo experiment, imaging was performed with a free-breathing 3D radial stack-of-stars UTE pulse sequence with a hard RF pulse. Each spoke was acquired in a center-out fashion to achieve an ultrashort echo time, as signal enhancement efficiency increases with shorter echo time [33]. The relevant imaging parameters include the following: TE/TR = 0.14/5 ms, flip angle = 8°, FOV = 496 × 496 × 250 mm^3^, matrix size = 256 × 256 × 30, the number of points in each spokes = 294 (consists of 24~48 pre-samples, 60 samples acquired during readout gradient ramping up), and spatial resolution = 1.94 × 1.94 × 8.3 mm^3^. For improving SNR, both hyperoxic and normoxic images were reconstructed at 1cm resolution along kz direction by non-uniform fast Fourier transform (NUFFT). Approximately 144,000 projections with golden angle increment (111.246°) were acquired to guarantee enough data for respiratory phase segmentation where 1600 views were used to segment average phase into four motion states.

### 2.3. Experiment Protocol and Materials

**Figure 1:**
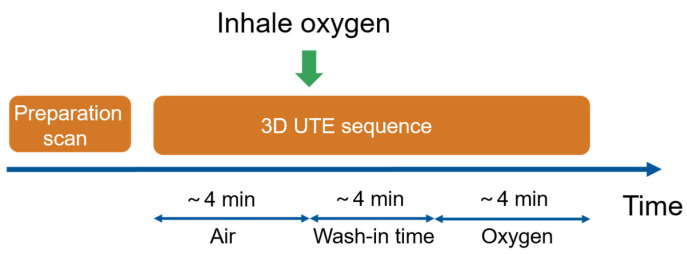
Flow chart of the experiment protocol using 3D UTE sequence with radial acquisition. Preparation scan is about 3 min for localization and shimming; hence, the total experiment time is approximate 15 min.

In traditional OE-MRI experiment, the imaging would be conducted twice for air and oxygen datasets acquisition. For golden angle radial technique, each successive spoke goes through the center of k-space and divides the largest remaining angular gap in half, making it possible to reconstruct image by grouping an arbitrary number of consecutive spokes. In our experiment, in order to reduce scan time, we conducted the imaging continuously during the whole experiment. The experiment protocol was shown in Figure 1. Forty-eight hundred views in total for each plane were collected. The first 1600 spokes were acquired with room air breath, which lasts about 4 min. After that, oxygen was delivered to the subject by non-rebreather face mask at 15 L/min flow rate [34], and the middle part in Figure 1 corresponds to the wash-in process, lasting about 4 min. During the wash-in process, oxygen would replace residual air in lungs gradually and consequently, the signal intensity increases with the partial pressure of oxygen. Normally, 3~5 min can ensure the signal intensity reaches a steady state. While oxygen concentration does not reach 100% in alveolar, OE MRI evaluates the lung function because the percent change in signal intensity between normoxia and hyperoxic breathing is representative of lung function, instead of the absolute value signal intensity or FiO2. Finally, oxygen-enhanced lung images were acquired by the last 1600 views. The total experiment time is approximately 12 min in addition to the preparation scan. 

Experiments were performed on a whole-body 1.5T clinical scanner (Xingaoyi Medical Equipment Co., Ltd., Ningbo, China) equipped with an eight-channel abdominal coil. Subjects are required to keep supine position and breathe uniformly. The non-rebreather face mask was used to provide high oxygen concentration. Subjects were monitored for the occurrence of adverse events. 

### 2.4. Respiratory Signal Extraction Method

The golden-angle radial acquisition ensures uniform coverage of k-space. The respiratory signal can be extracted from the k-space data. Following previous literature [28,35,36,37], there are two algorithmic stages for respiratory signal detection. With center-out acquisition style, the first point of each spoke was selected to conduct one-dimensional (1D) fast Fourier transform (FFT) to generate projection profiles of all coils. Although signal enhancement is much lower than other contras agents (Gadolinium-DTPA), normalization of signal intensity is also necessary to alleviate signal fluctuation by oxygen enhancement. Moreover, the amplitude change of projection profiles consists of both respiratory motion and cardiac motion, which have almost regular frequency of 0.1–0.5 Hz and 0.6–3.0 Hz, respectively. Hence, the respiratory motion signal is able to be separated from the mixed-signal by filtering according to the frequency bands. Subsequently, principal component analysis (PCA) was used along with each coil for selecting the peak of projection profiles that is within the range of respiratory motion frequency and consequently find the ‘good’ coils as the representation [36]. Secondly, signals vary with coil location. The most common signal among the ‘good’ coil component was found as the representative respiratory signal [38]. 

**Figure 2:**
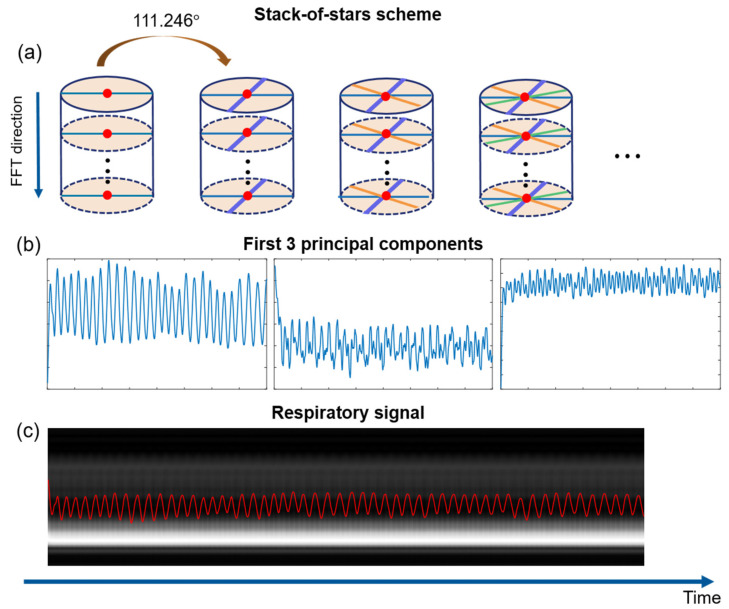
Respiratory motion extraction method. (**a**) 3D stack-of-stars acquisition scheme with gold angle (111.246°) rotation. One dimension Fourier transform along kz direction was conducted on the center point of each spoke (red points), generating projection profile (shown in (**c**)). (**b**) First three principal components were extracted from projection profile by PCA algorithm. (**c**) The first component was selected as respiratory signal superimposed on the corresponding projection profile.

Image reconstruction was performed offline in the Matlab programming environment. The aim of our study is to investigate the signal enhancement of the lung images with pure oxygen administration, so the normoxia and hyperoxic images were reconstructed based on the extracted respiratory signal, respectively, and the dynamic process is out of our study. Therefore, there is no need to subtract the oscillation resulting from signal change when extracting respiratory motion signal from the whole data set. Figure 2 displays the process of respiratory motion extraction. 

### 2.5. Phase-Resolved Pulmonary Imaging

Since the golden-angleacquisition scheme allows the retrospective sorting of k-space data, the dataset can be subdivided into arbitrary motion states. The common method for phase segmentation is based on amplitude of extracted respiratory signal, where the uniform binning uses the static threshold to select the motion states, and optimized binning uses an adaptive threshold. This approach performs well on the application of dynamic contrast enhancement (DCE) MRI. Nevertheless, for OE MRI, both air and oxygen volumes need to be processed so that a single threshold is not suitable to both as there may be different spokes for phase reconstruction of two data sets.

**Figure 3:**
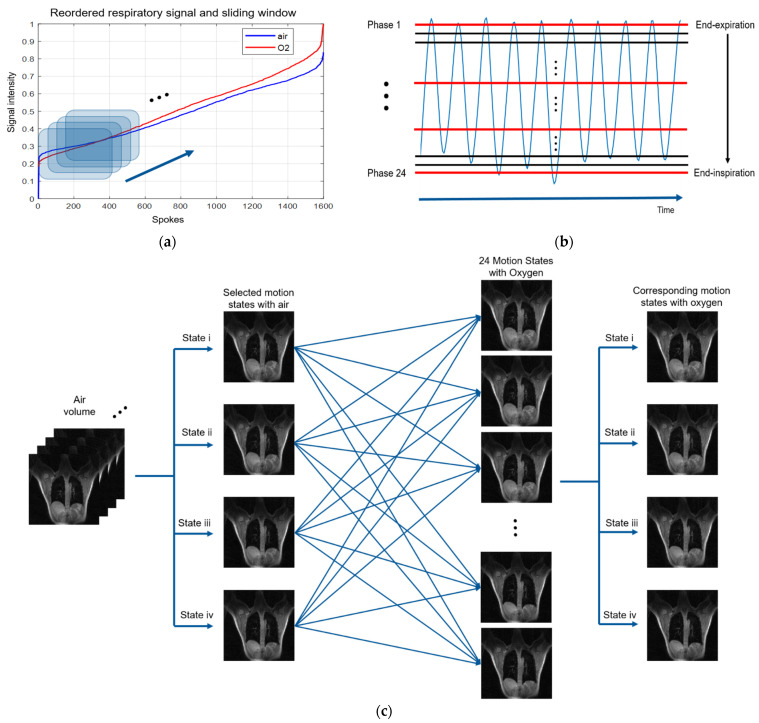
Respiratory phase segmentation. (**a**) Reordered respiratory signals during breathing room air and oxygen. The shadow squares are sliding window containing 450 spokes, reconstructing 24 respiratory states from the end of inspiration to the end of expiration. (**b**) Four representative respiratory states (highlighted in red lines) were selected from these 24 respiratory phases. (**c**) Graphical diagram of air-oxygen respiratory matching states selection. Four respiratory states were selected from air image volume first. The matching respiratory states of oxygen volume were selected manually by comparing with the position of diaphragm. State i~state iv represent the selected respiratory states with air (the second column on the left) and oxygen (the right column).

For improving the accuracy of respiratory phase segmentation, sliding window technique was applied in this study. Technically, the minimum time resolution is $N_{z}\times TR$. For example, the second motion state can be reconstructed by dropping the first spoke, replacing it with the new view [39]. Figure 3 displays the respiratory phase segmentation technique used in this paper. Specifically, all collected stack-of-stars UTE spokes are resorted by its extracted respiratory signal amplitude, corresponding to respiratory cycle, as shown in Figure 3a. In this study, both air and oxygen datasets were reconstructed into 24 respiratory phases by sliding window technique with 50 spokes internal on each phase using the resorted 1600 spokes. In other words, the reconstruction window would slide 50 spokes along respiratory signal when reconstructing next respiratory phase. For example, the first respiratory phase is reconstructed by 1st~450th spokes, then the second respiratory phase is reconstructed by 51st~500th spokes until the last phase is reconstructed by 1151st~1600th spokes. This method ensured that the number of spokes of each state is the same. Nevertheless, respiratory rate and inflation volume may be slightly different between air and oxygen acquisition, shown as the blue and red lines in Figure 3a. To make a fair comparison, the four respiratory states with oxygen were selected manually based on comparing the diaphragm position. Figure 3b demonstrates the 24 respiratory phases from end-expiration to end-inspiration after segmentation, including four selected phases highlighted in red lines. 

### 2.6. Image Analysis

**Figure 4:**
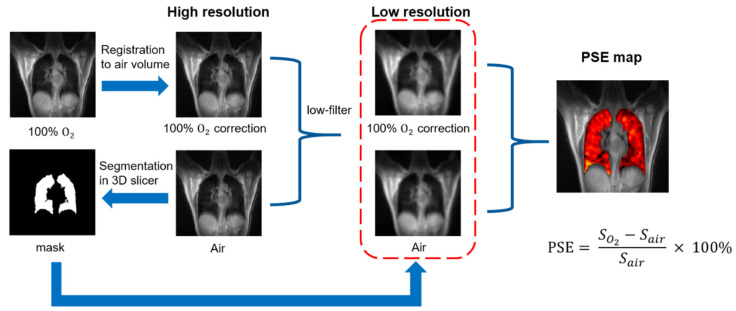
A pictorial representation of image analysis. First, the oxygen volume image was registered to the corresponding air volume image. High-resolution hyperoxic images were segmented semi-automatically by 3D Slicer to produce a binary lung mask. The low-resolution volumes were obtained by applying Gaussian low-pass filter on k-space. PSE map was generated by Equation (1), superimposed on a high-resolution air image.

The dataset was subdivided into two volumes based on oxygen concentration. Non-phase-resolved normoxia and hyperoxic image volumes were reconstructed by 800 spokes at the beginning and last part of the whole dataset, ensuring enough time for oxygen wash-in. Phase-resolved normoxia and hyperoxic images were reconstructed as explained in Section 2.4. Figure 4 is the data processing pipeline. The hyperoxic image volume was registered to the corresponding normoxia volume by non-rigid demons registration algorithms [40]. Segmentation was semi-automatically completed in 3D slicer (https://www.slicer.org/, accessed on 29 October 2021), excluding aorta and blur near the diaphragm. Signal enhancement can be evaluated by PSE or mean PSE (MPSE), which is calculated on pixel level by dividing signal intensity of normoxia image from the signal difference of both images. Before PSE calculation, the high-resolution images were filtered to low resolution (1 cm × 1 cm) in each plane by Gaussian low-pass filter to improve SNR. 

PSE maps were calculated using Equation (1), where $S_{O_{2}}$ and $S_{air}$ represent the signal intensity of the hyperoxic and normoxic images, respectively. Both PSE and MPSE of each partition on all subjects were calculated to investigate the gravity effect on the distribution of signal enhancement. Repetitive experiments were also performed. PSE distribution curve of each state was fitted based on the histogram of PSE map.

(1) $$\mathrm{PSE}=\frac{S_{O_{2}}-S_{air}}{S_{air}}\times 100\%$$

## 3. Results

### 3.1. Simulation Results

**Figure 5:**
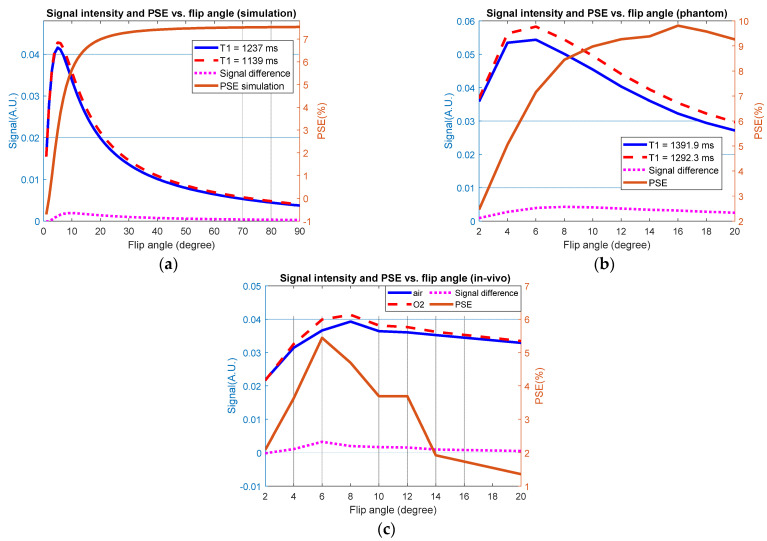
Signal intensity and PSE with variable flip angle: (**a**) Bloch simulation using T1/T2* reported in previous literature. (**b**) Experiment results with homemade T1 phantoms. (**c**) Experiment results on a healthy human. All PSE was calculated by Equation (1).

To optimize parameters of UTE sequence, Bloch simulation was conducted before phantom and in vivo experiments. Figure 5a shows the simulated signal intensity and PSE of two tissues with different T1/T2*. The maximum signal intensity of both tissues occurs at 5° of flip angle, consistent with the theoretic result of Ernst angle, while the MPSE signal monotonically increases with flip angle, reaching almost 8% finally. Even so, lower SNR may affect the accuracy of signal enhancement calculation if the signal intensity is too low. There is a cross point on 9° hence, according to the simulation results, the flip angle range of 8°~10° could be a tradeoff between image quality and enhancement. Figure 5b displays the experiment results with flip angle range of 2~20° on homemade phantoms. A similar trend was founded on both signal intensity and PSE. The cross point occurs at 8°. In vivo experiment was conducted on a subject with flip angle changing from 2° to 20°. There are five slices selected from anterior to posterior. The final percent signal enhancement was the average of all slices. The in vivo experiment showed a quite different trend on PSE curve, especially in larger flip angle where the PSE reduced rapidly. The maximum signal intensity of both air and oxygen volumes is at 8°, while the maximum signal enhancement is 5.44% where flip angle is 6°. 

Since lung tissue density is much lower than other organs and air–tissue contact affects negatively SNR, it is important to improve signal intensity when imaging lungs. Considering simulation and phantom experiment results only, the flip angle (9° and 8°, respectively) where the maximum difference occurred may be the best choice. Nevertheless, Figure 5c illustrates that although the PSE and maximum difference at 6° is higher than those at 8°, the signal intensity is higher when flip angle equals 8°. Additionally, the flip angle used in previous literature is 8°, closer to simulation and phantom results [20,26,41]. Considering all results in Figure 5, we think the flip angle of 8° is reasonable, balancing the signal intensity and MPSE. This is the tradeoff of all results. Therefore, we selected 8° as used flip angle in our subsequent experiments. 

### 3.2. Global Measurements

**Figure 6:**
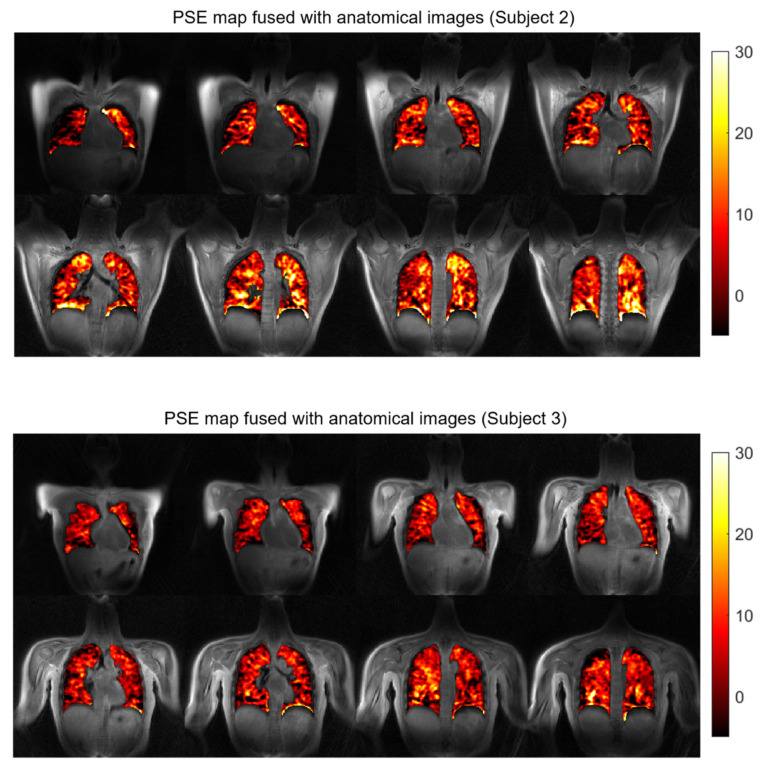
PSE maps of eight slices on two subjects. Both datasets show the obvious increase on PSE distribution from anterior to posterior region.

**Figure 7:**
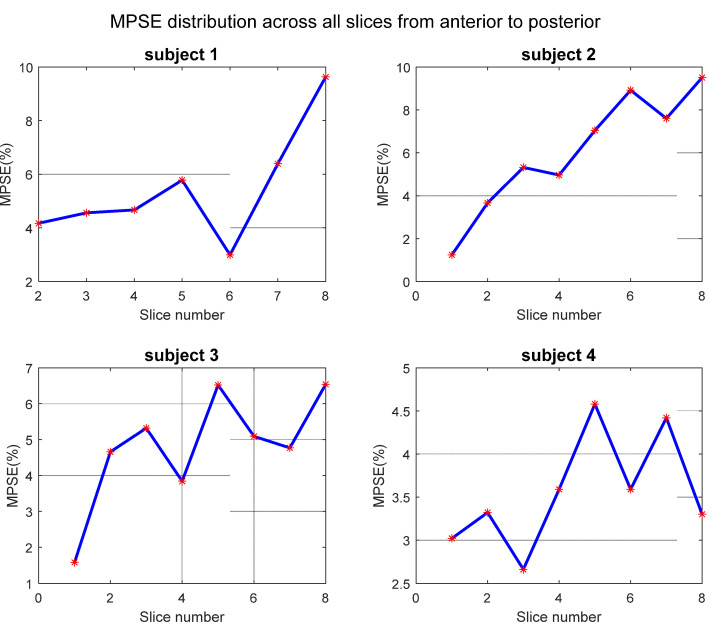
MPSE of four subjects on eight slices without respiratory motion segmentation. There are seven slices for subject 1 and 8 slices for all other subjects.

Non-phase-resolved Hyperoxic and normoxic images of average phase were reconstructed from the first 800 spokes and last 800 spokes, respectively. Eight planes were selected to cover lung region from anterior to posterior. Two representative subjects’ results are displayed in Figure 6, illustrating increased trend of PSE and ventilation defect area that showed as the black regions in PSE map. Since no respiratory motion segmentation is applied, soft blur is obvious in the area near the diaphragm which has been excluded when calculating PSE. The higher PSE occurs on the region near vessels and diaphragm. It could be explained that the oxygenated blood also made contribution on signal enhancement. In terms of the diaphragm area, the respiratory motion also has effects on mask extraction. Figure 7 shows mean PSE of each slice of all subjects from anterior to posterior region. The last four slices were regarded as posterior area, while the first three or four slices were in anterior part. Although there is fluctuation from the front to back, the results suggest a general increase trend on PSE. The average PSE in the posterior area is increased by 38.75%, 117.85%, 48.83%, 26.21%, respectively, higher than those reported in previous literature [20]. The possible explanation for this phenomenon could be the posterior area is easy to be affected by the respiratory motion, resulting in more severe motion average effects. Furthermore, gravity and diffusion may also affect the gas distribution in lung tissue because there is plenty of space for oxygen diffusion in alveoli [42,43]. The data shown in Figure 6 and Figure 7 demonstrate good consistency. 

Over four subjects, the whole lung MPSE varied between 3.56% and 6.03% (4.96 ± 1.06%), consistent with Bloch simulation and results displayed in Figure 5c. Table 1 is the summary of the demographic information and pulmonary function test of subjects. 

### 3.3. Phase-Resolved Image and PSE Map

**Figure 8:**
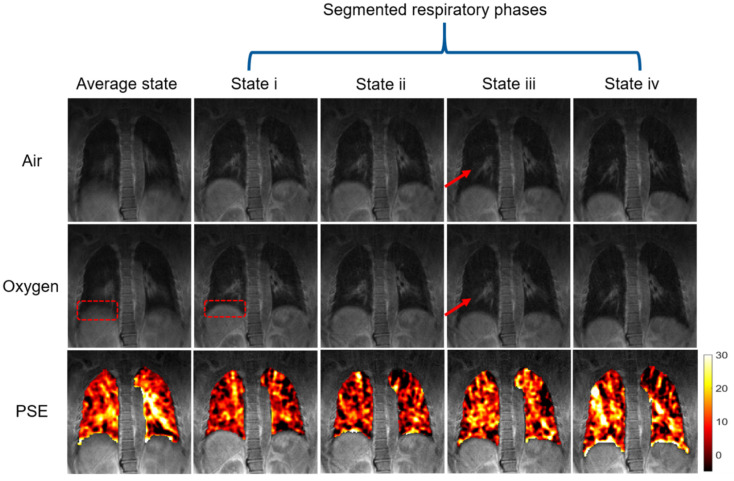
Phase-resolved lung images and corresponding PSE map of subject 1. The first column represents image without phase segmentation, where blur on diaphragm was highlighted by red dash square. The corresponding phase-resolved images are listed in columns 2 to 5 from end-expiratory to end-inspiratory. The tissues’ signal highlighted by red arrows in oxygen image are stronger than that in air image. The bottom row is PSE map fused on the same coronal plane with a specific PSE value of 9.63%, 5.0%, 4.12%, 7.87%, and 8.63%.

Figure 8 displays both air and oxygen images processed by phase segmentation technique as well as corresponding PSE maps superimposed on high-resolution air image. Four states from end-expiration (state i) to end-inspiration (state iv) were selected from 24 air–oxygen image pairs reconstructed by the sliding window technique based on the diaphragm position. The red dash square on diaphragm highlights that phase-resolved technique is able to eliminate respiratory motion related blurring. Besides, compared to the average state, the vessels in segmented respiratory phases are much clearer. The arrow highlight that the signal displayed an obvious increase on vessels of hyperoxic image at middle respiratory state, which may be significant for some lung diseases detection in the future. However, the mean PSE is largest in the average state, reaching 9.63%, higher than segmented motion states. 

**Figure 9:**
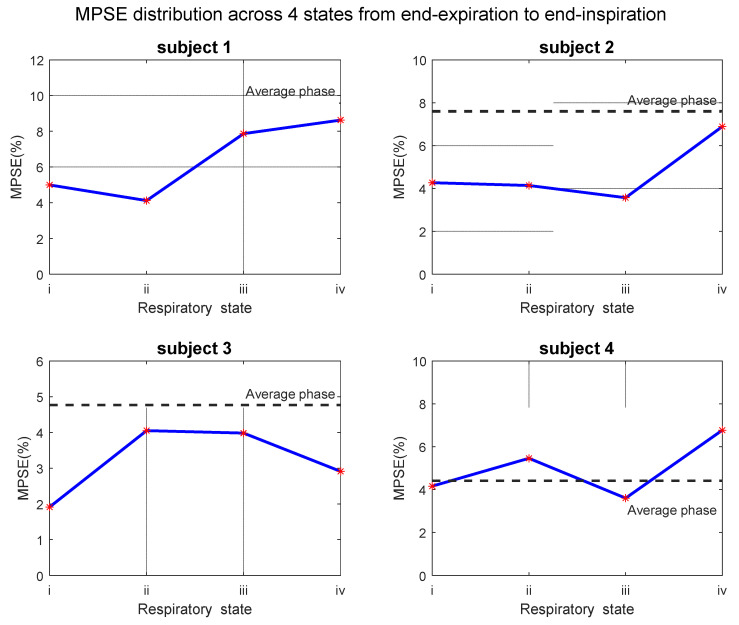
Segmented respiratory states MPSE of four subjects on the same plane which placed near back part where the respiratory motion effect is serious than anterior region. The gray dash line represents the MPSE value of the average phase. The horizontal axis in each sub-diagram means respiratory states from end-expiration to end-inspiration (i to iv). The PSE map of each state of subject 1 corresponds to the bottom row in Figure 8.

Figure 9 shows the mean PSE of each segmented phase of all four subjects from end-expiration to end-inspiration. The MPSE of end-inspiration state (state iv) is higher than the value at the end of expiration (state i), statistically increasing 72.6%, 61.4%, 52.4%, and 62.7%. On the other hand, MPSE of middle states is between values of both respiratory ends except for subject 3, where the MPSE of both middle states are higher than end states. For subjects 2~4, there is slight difference on MPSE between both middle respiratory phases. The large difference of MPSE on four segmented states was observed on subject 1. This may be due to the less motion between the first two states. It can be found that state ii is closer to state i than state iii in Figure 8, resulting in similar MPSE between the first two states. Another observation is that MPSE values of segmented phases are lower than the average state apart from subject 4. The reason for this phenomenon will be discussed in detail in Section 4.

### 3.4. Test–Retest Repeatability

**Figure 10:**
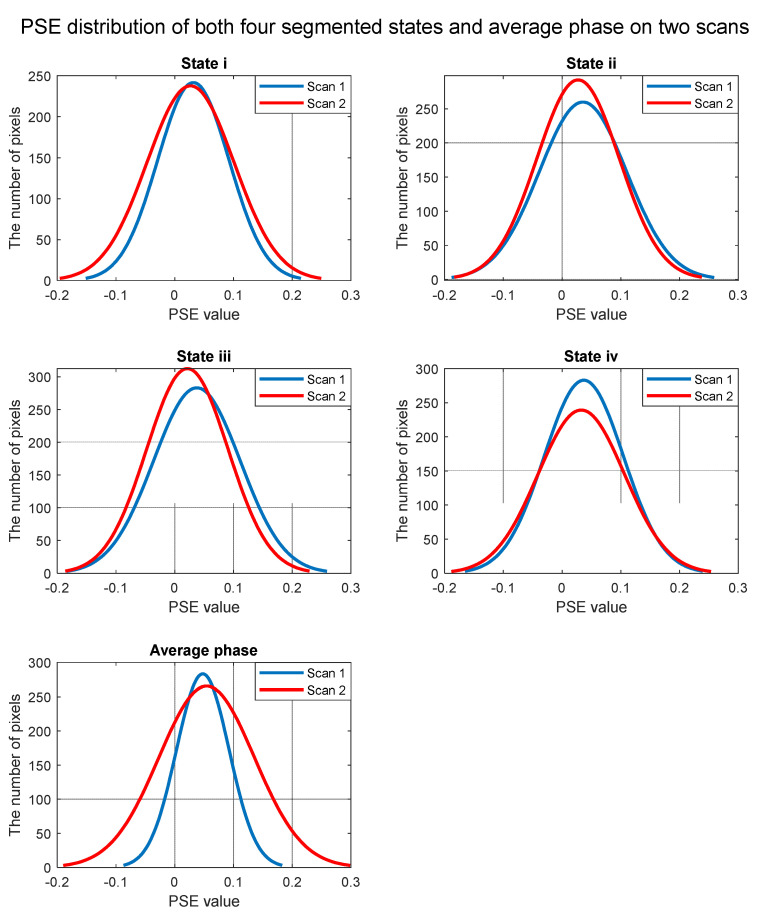
PSE distribution of both average phase and four segmented states on two scans that performed within two weeks.

Repeatability was tested by conducting the experiment on same subjects on different days. Coefficient of Variation (CV) was calculated to evaluate the repeatability of the PSE calculation. States i, iii and iv have relatively better repeatability with lower coefficient of variation (1.44%, 8.27%, and 5.57%, respectively), compared with that of the average phase and state ii (19.1%, 21.5%, respectively). Figure 10 is the PSE distribution curve of both segmented phases and average phase. For segmented phases, reasonable difference on PSE distribution was observed between two scans. By contrast, the average phase demonstrates larger difference on PSE distribution. However, although the peak value of PSE from state ii and state iv is similar between two repetition scans, the distribution is quite similar, respectively. The possible reason may be that different masks extracted by image segmentation were used for two scans’ datasets, which leads to different total number of pixels plotted in histogram. Overall, the repeatability of segmented states is acceptable and better than average phase, but not excellent compared with current publications [41,44]. The reason will be analyzed in the discussion section. 

## 4. Discussion

We have demonstrated the feasibility of using 3D phase-resolved technique to investigate the oxygen-enhanced lung MRI. 3D radial UTE sequence with the stack-of-stars scheme was used in our study to generate 3D images of both air and pure oxygen breathing in healthy human. Since each spoke collects the center point of k-space, radial acquisition scheme has the inherent average effect, which enables it to be insensitive to respiratory and cardiac motion. Compared to the 2D IR-HASTE sequence, 3D UTE allows free-breathing and interpolation in kz direction allows us to reconstruct more planes with higher resolution [45]. Despite lower MPSE value, UTE MRI provides more details of lung tissue and vessels than IR-HASTE sequence. PSE maps of variable planes were investigated as well, as shown in Figure 6. All subjects’ results show that the PSE in posterior area is higher than that in anterior region. The possible reason could be that the gravity causes more gas to gather at the back area when volunteer is in supine position during experiment [20,42]. Another explanation could be the various vascular distribution as the oxygen-enhanced MRI is the combined results of ventilation, diffusion and perfusion. On the other hand, we also found that the respiratory motion effect is smaller in the anterior region as there is less soft blur. 

Since lung density and volume are changing while breathing, it is difficult to lock a specific state of a respiratory cycle. 3D UTE images in the average phase have residual blurring near diaphragm, as displayed in Figure 8. The common method is to use the respiratory gate and ECG gate to collect signals at the end of expiration [24,25] and throw away other signals. In our study, a self-gated method was used to separate the respiratory phases so that there are no dropped signals and more motion states could be extracted. Sliding window technique was applied to reconstruct 24 phases from the end of expiration to the end of inspiration, in which four represented phases including both end states and two middle states were selected for the final calculation based on the diaphragm position. A significant advantage of this approach is that segmented phase pair is much more accurately co-registered spatially than the traditional method based on the threshold of respiratory signal amplitude. 

We also investigated a new data acquisition protocol in this study. For traditional oxygen-enhanced MRI experiments, the same image volume should be collected twice, which increases the scan time, especially for phase-resolved imaging which needs more spokes for visualizing separated phases. The sequence was conducted continuously throughout the whole experiment, including the air, wash-in process and 100% oxygen part. Therefore, simplified experiment operation and zero time interval among every part also reduced the experiment time, consequently bringing less bulk motion that is likely exacerbated by relatively long examination times used in traditional protocol. Since radial acquisition allows the reconstruction with arbitrary spoke groups, the continuous protocol with sliding window reconstruction also could be used to investigate the wash-in and wash-out time of oxygen-enhanced MRI, which is beyond the scope of the present work but would be investigated in the future study. 

Compared with average phase image, the phase-resolved MRI images provide clearer details, displayed in Figure 8. For the patients with great breathing range, severe soft blurs occur resulting from respiratory motion. Phase-resolved technique could solve this problem by locking the respiratory phase. Phase-resolved OE-MRI shows excellent performance on illustrating lung tissue details in hyperoxic images, such as diaphragm region and vessels. However, the PSE maps calculated by phase-resolved images is lower than the average phase. There are two possible reasons for this phenomenon. First, signal enhancement of oxygen-enhanced MRI is the combined results of gas ventilation, diffusion and perfusion. Middle state of a respiratory cycle is much faster than end states so that there is less time for oxygen-gas exchange. Second, the middle states introduced in this study were the combination of inhalation and exhalation processes. Blood also contributes signal enhancement by two different mechanisms, namely the paramagnetic property of deoxyhemoglobin and molecular oxygen [10]. During the middle part of inhalation, there is deoxyhemoglobin in vessels, and fresh oxygen goes into lungs for gas exchange. At the same time, oxygen also dissolved into blood, shortening the T1 relaxation time of the pulmonary venous blood and consequently contributing to the signal enhancement as well [46]. Conversely, for the exhalation process, dissolved molecular oxygen has flowed away from lungs so that there may be less signal enhancement. Future studies should be conducted to investigate the motion states of inhalation and exhalation, respectively.

The MPSE of end-respiration is higher than other states, which is consistent with the results reported by previous authors [27]. This could be explained as there is most oxygen in lung tissue as well as oxygenated blood. The repeatability test demonstrated reasonable performance on separated phase performance. 

This feasibility study still has several limitations. First, the volunteer group only includes healthy subjects. More investigations are needed to evaluate the clinical performance on patients with variable lung diseases. Secondly, the work focuses exclusively on the decomposition of respiratory motion as lung volume is affected mainly by respiration. On the other hand, the cardiac motion also influences the signal enhancement when breathing pure oxygen due to the enhancement by oxygenated blood. Fourier decomposition technique could be used in the future to evaluated the ventilation and perfusion influence separately [47]. Thirdly, Demons algorithm may not be the best registration algorithm as the greyscale-based theory may reduce the difference between two images, leading to worse repeatability. Although the image mismatch was avoided as much as possible by conducting scan without stops within a short time, the influence may not be ignored and worth trying with more efficient algorithm, such as locally deformable registration methods [44]. Finally, to reduce scan time, we reconstructed images using under-sampling data without any acceleration algorithm, leading to relatively lower image quality; for example, 400 spokes were used to reconstruct resolved phase images, whereas Nyquist Theorem requires 800 spokes for 256 × 256 metrics on the center-out trajectory. 

The technique of phase-resolved oxygen-enhanced lung imaging could be used to investigate the gas exchange on patients by monitoring PSE map of middle states. Wash-in and wash-out time constants of oxygen respiration are also able to be measured using sliding window reconstruction based on radial acquisition scheme. 

## 5. Conclusions

In this paper, we have demonstrated the feasibility of generating reasonable hyperoxic images and PSE maps at different respiratory phases using self-gated free-breathing stack-of-stars UTE sequence. Compared to traditionally free-breathing images, phase-resolved oxygen-enhanced MRI provided more details on lung vessels and sharp edges on diaphragm. Additionally, the enhancement is easier to be observed on the anatomic image on separated motion states. The modified experiment protocol improved the efficiency of data collection, reducing scan time. Sliding window based reconstruction provided accurately segmented normoxia and hyperoxic image pairs. Therefore, the application of phase-resolved oxygen-enhanced imaging could be useful in tracking diseases related to ventilation. We also statistically confirmed that the MPSE increases from anterior to posterior region. Since this feasibility study only includes healthy volunteers, further study is needed to investigate the PSE map on patients at different respiratory phases.

## Figures and Tables

**Table 1:** Subject demographic information, as well as median PSE of masked OE volumes.

Information	Subject 1	Subject 2	Subject 3	Subject 4	Mean ± Std
Age	46	28	29	28	32.8 ± 8.8
Height (cm)	170	178	160	172	170 ± 7.5
Ethnicity	North East Asian	North East Asian	North East Asian	North East Asian	-
FEV1 (L/%) ^1^	4.68/126.8	4.54/103.2	3.6/117.1	5.25/128.8	-
FVC (L/%) ^1^	4.83/106.7	4.54/85.9	3.81/106.6	5.25/107.9	-
FEF2575 (L/s/%) ^1^	7.56/205.1	7.85/173.1	4.05/123.9	9.25/216.1	-
FEF75 (L/s/%) ^1^	3.39/212.7	4.86/215.2	2.09/128.3	6.29/299.4	-
FEV1/FVC	0.97/118.5	1.00/119.4	0.95/109.6	1.00/118.8	-
MPSE (%)	5.46	6.03	4.79	3.56	4.96 ± 1.06

^1^ Spirometer data include measured value/predicted percentage value.

## Data Availability

The data presented in this study are available on request from the corresponding author. The data are not publicly available due to ethical limitation.
